# Duodenal Hemangioma as a Rare Cause of Gastrointestinal Bleeding: A Case Report and Literature Review

**DOI:** 10.7759/cureus.43293

**Published:** 2023-08-10

**Authors:** Osama N Dukmak, Mohammed Ayyad, Mahmoud Gabajah, Fida’ Dabbas, Ayman Budair, Mohammad Emar, Mohamed Maraqa, Fahmi Joubran

**Affiliations:** 1 College of Medicine, Al-Quds University, Jerusalem, PSE; 2 Internal Medicine, Al-Quds University, Jerusalem, PSE; 3 Surgery, Al-Quds University, Jerusalem, PSE; 4 General Surgery, Al-Quds University, Jerusalem, PSE; 5 General Surgery, Al-Ahli Hospital, Jerusalem, PSE

**Keywords:** venous hemangioma, vascular malformation, endoscopic resection, sclerotherapy, gastrointestinal bleeding, duodenal hemangioma

## Abstract

Duodenal hemangiomas are benign vascular tumors caused by haphazard vascular proliferation within the duodenal wall. Although rare, duodenal hemangiomas could lead to rapidly progressive life-threatening gastrointestinal (GI) bleeding that requires urgent intervention. The diagnosis of duodenal hemangioma often requires direct visualization of the lesion either endoscopically or surgically, as well as histopathological examination. Treatment options include endoscopic resection, laser coagulation, sclerotherapy, or in a specific subset of patients, open or laparoscopic surgical intervention.

We herein report a case of a 46-year-old female presenting with signs and symptoms of chronic GI bleeding. The patient underwent upper endoscopy and was found to have an ulcerated mass in the proximal duodenum consistent with the diagnosis of duodenal hemangioma. This case highlights the importance of including duodenal hemangioma in the differential of upper GI bleeding. It also underscores the significance of surgical intervention in treating duodenal hemangioma, as well as the crucial role of employing endoscopy in the diagnostic and therapeutic management of this condition.

## Introduction

Hemangiomas are benign vascular tumors that typically originate from the submucosal layer of blood vessels [1]. They tend to develop on the skin and internal organs such as the liver. The occurrence of hemangiomas within the gastrointestinal (GI) tract is extremely rare, as these tumors typically make up less than 0.05% of all GI tumors, and 7-10% of small bowel neoplasms [2]. Additionally, they are considered a very uncommon cause of GI bleeding. Although these malformations could occur at any location along the GI tract, the small intestines are the most common site for hemangiomas to develop [3]. Duodenal hemangiomas, on the other hand, are typically discovered incidentally during endoscopic procedures in asymptomatic patients [4]. Conversely, symptoms could occur and these include fatigue and dyspnea related to anemia, abdominal pain, hematemesis and melena, mechanical bowel obstruction, intussusception, and perforation [4]. Interestingly, the presence of multiple lesions along the GI tract is often associated with similar lesions in solid organs such as the liver [5]. Although minimally invasive endoscopic procedures are gaining popularity in the treatment of hemangiomas, larger and persistent lesions still require resection with an open surgical technique [2].

We describe a case of a 46-year-old patient presenting with weakness, fatigue, and melena. Endoscopy revealed the presence of an ulcerated oozing duodenal hemangioma. The tumor was resected using an open surgical technique leading to the complete resolution of the patient's condition.

## Case presentation

A 46-year-old female presented to the emergency department complaining of generalized weakness for one-week duration. Her past medical history was significant for hypertension and heart failure with a preserved ejection fraction (EF) of 50%, both of which were diagnosed seven months ago. Her medications included candesartan, furosemide, and multivitamins. On physical examination, the patient looked pale and fatigued. She was conscious, alert, and oriented to time, place, person, and situation. Her abdomen was soft with no palpable masses or organomegaly.

On further questioning, the patient reported generalized weakness and fatigue that developed sub-acutely over the course of a few days. She also had palpitations, bilateral lower limb myalgia, and tar-colored stools for the past month. During that time, laboratory investigations including a complete blood count (CBC) revealed a hemoglobin of 6 g/dL (N=12.1-15.1 g/dL). Subsequently, the patient was admitted to the hospital and had two packed red blood cell transfusions. The patient's symptoms and hemoglobin subsequently improved leading to discharge five days post-admission.

**Figure 1:**
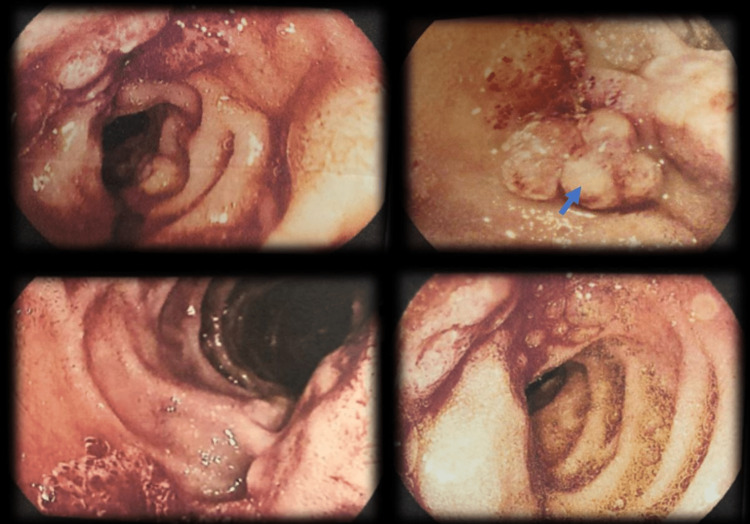
Multiple endoscopic views showing an ulcerated oozing post-bulbar duodenal mass suggestive of a duodenal hemangioma (arrow).

Three days post-discharge, the patient developed hematemesis of dark-colored blood, which was intermittent and estimated as one full cup in amount. Furthermore, the patient complained of recurrent generalized weakness, fatigue, and anorexia. She was readmitted to the hospital and stabilized with aggressive intravenous (IV) fluid therapy and proton pump inhibitors. A complete metabolic workup including electrolytes, liver, and kidney function tests was unremarkable. Abdominal computed tomography (CT) with IV contrast revealed focal polypoidal non-enhancing wall thickening seen at the second part of the duodenum, along with multiple non-enhancing cystic lymph nodes seen in the retrocaval, para-aortic, and the right peri-renal spaces. In addition, a small homogenous liver mass was detected. Put together, the findings were suspicious for a duodenal malignancy with metastatic lymphadenopathy. Consequently, an upper endoscopy was performed and revealed a 2 cm ulcerated actively bleeding mass located on the post-bulbar lateral wall of the duodenum opposite to the ampulla of Vater (Figure 1).

**Figure 2:**
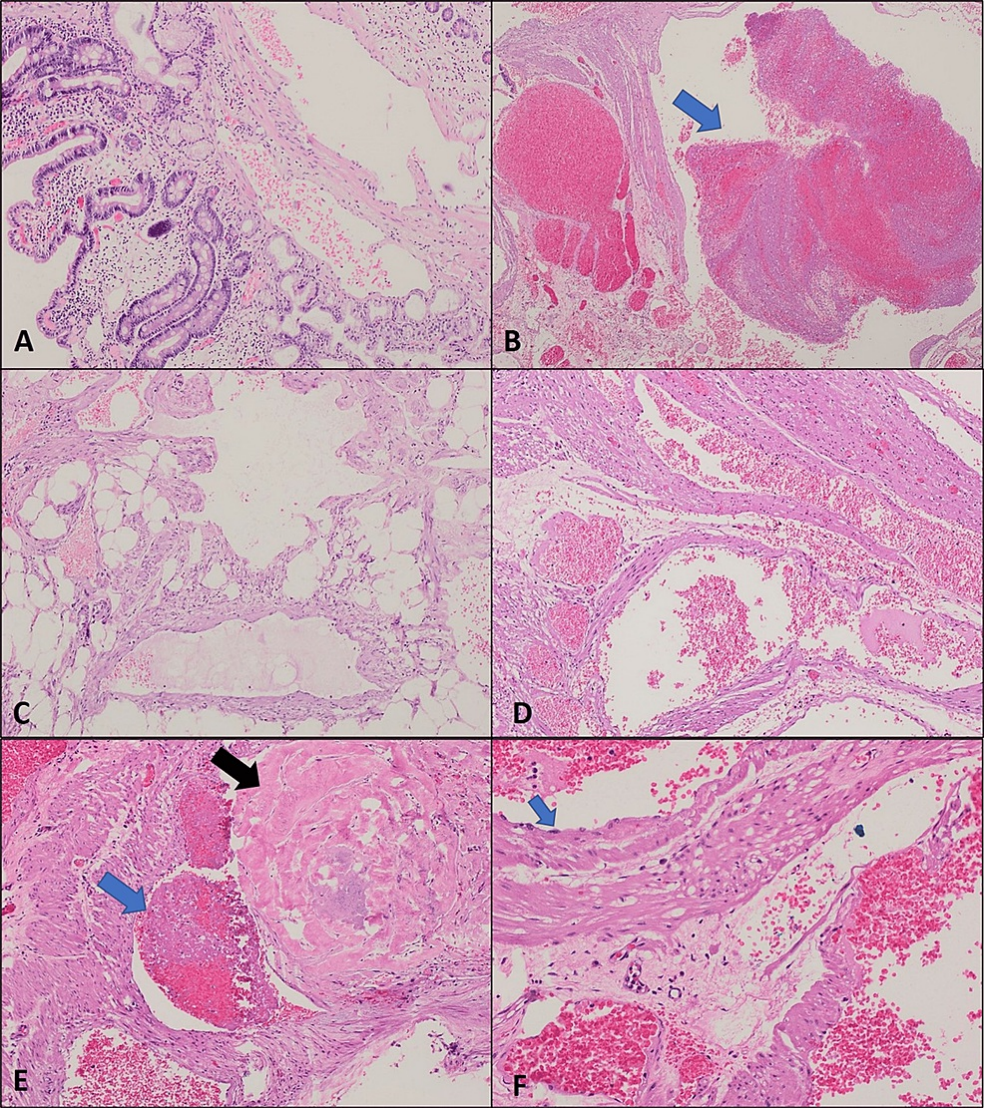
Histopathological slides showing findings consistent with a venous hemangioma/malformation of the duodenal wall. Duodenal specimen showing submucosal dilated vascular spaces (H&E, ×4) (A); multiple resected duodenal specimens (B-E), low power view of the duodenal wall (muscularis propria) showing multiple dilated vascular spaces with the presence of a recent thrombus (arrow) (H&E, ×4) (B); the presence of the vascular lesion within adipose tissue (H&E, ×4) (C); thick-walled blood vessels (H&E, ×4); recent (blue arrow) and old thrombi with early calcification (black arrow) (H&E, ×10) (E); and thick muscular wall and bland endothelial lining (arrow) (H&E, ×10) (F).

On the second-day post-admission, a right subclavian central line was applied and the patient was started on total parenteral nutrition (TPN). Input-output charts were recorded along with an order to repeat CBC every 12 hours to monitor for deterioration. On the fifth day post-admission, a pre-operative assessment was done and two units of packed RBCs were transfused. The patient subsequently underwent explorative laparotomy, during which a small firm non-mobile lesion was found at the antimesenteric border of the second part of the duodenum measuring about 1×2 cm. Small preduodenal cystic lymph nodes were also observed. The surgeon then performed wedge resection of the duodenal mass and the preduodenal lymph nodes, after which an abdominal drain at Morrison's pouch was inserted. The specimens resected were sent for histological examination, which confirmed a venous hemangioma involving the submucosa, muscularis propria, and surrounding adipose tissue (Figures 2A-2F). Additionally, the lymph node specimen showed benign histological findings.

Postoperatively, the patient was transferred to the surgical ward for observation. She was kept on nothing-by-mouth (NPO) status. A nasogastric (NG) tube was inserted, and IV fluids along with antibiotics and analgesics were administered.

The patient continued to improve leading up to the removal of the central line and the NG tube. Oral feedings were then attempted with good tolerance. She was discharged with an abdominal drain on day six post-operatively in good general condition with no complaints. The patient was counseled to follow-up at an outpatient clinic and showed marked improvement over the next few months with no signs of recurrence.

## Discussion

Intestinal hemangioma is a rare benign overgrowth of the submucosal vessels characterized by vascular and endothelial proliferation of the submucosal vascular bed in the intestine. It most commonly presents in young adults in their 20s, however, there are a few cases reported in the pediatric population [6]. Conversely, our patient presented at the age of 46 years, which is relatively uncommon. Additionally, the tumor accounts for only 0.3% of all gastrointestinal tumors, with the duodenum being an unusual location for this neoplasm [7]. This often leads to difficulty and delay in the diagnosis of the condition.

On histopathological examination, small intestine hemangioma is classified into three types according to the type of vessel involved. These include cavernous hemangioma, which is the most common type, as well as capillary and mixed types [6]. The histopathological examination of the mass in our patient revealed a cavernous type hemangioma of venous origin, which is considered an uncommon variant. Two cases out of 11 found in the literature involved venous circulation as shown in Table 1 [2,8-17]. The first case of duodenal hemangioma was reported in 1978 and a total of 23 cases were reported during the past 44 years [6]. Between 1978 and 2008, only 21 cases of duodenal hemangioma were reported in the literature, of those, cavernous hemangiomas accounted for just over half of the cases. Between 2008 and 2019, two cases of duodenal hemangiomas were reported, including one case of cavernous hemangioma [6,18].

**Table 1:** Summary of duodenal hemangioma cases reported between 2008 and 2021. M: male; F: female; EMR: endoscopic mucosal resection; APC: argon plasma coagulation; IDA: iron deficiency anemia

Case reports	No. of cases	Gender/age (years)	Complaint/ presentation	Location	Single/multiple	Treatment	Pathology
Present case	1	F/46 years	Generalized weakness/fatigue	Second part of the duodenum	Multiple	Surgical resection	Cavernous (venous)
Hu et al. [2]	1	F/24 years	Melena/fatigue	Ileum	Single	Surgical resection	Cavernous
Takase et al. [8]	2	F/62 years; M/52 years	Melena; malaise and melena	Jejunum; ileum	Single/single	Laparoscopic resection; laparoscopic resection	Cavernous (capillary)
Heo et al. [9]	1	M/38 years	Melena	Jejunum	Single	Surgical resection	Cavernous (venous)
Ocampo et al. [10]	1	M/29 years	Chronic anemia	Ileum	Single	Laparoscopic resection	Capillary
Chen et al. [11]	1	F/27 years	Chronic IDA	Ileum	Single	Laparoscopic resection	Cavernous
Fernandes et al. [12]	1	F/56 years	Hematochezia, syncope	Ileum	Single	Surgical resection	Cavernous
Pera et al. [13]	1	M/16 years	Fatigue, IDA, palpitation	Jejunum	Single	Surgical resection	Cavernous
Easler et al. [14]	1	M/71 years	Anemia/melena	Jejunum	Single	EMR	Cavernous
Elias and Toubia [15]	1	M/30 years	IDA/blood in stool	Jejunum	Multiple	Surgical resection	Cavernous (venous)
Shibuya et al. [16]	1	M/74 years	Melena	Jejunum	Single	EMR	Capillary
Ng et al. [17]	1	F/20 years	Anemia	Terminal ileum	Multiple	APC	-

The clinical presentation of duodenal hemangioma varies broadly. The mass can be discovered incidentally during the performance of an endoscopic procedure, or have non-specific non-localizing signs and symptoms. Similar to our patient, most cases typically present with signs and symptoms related to chronic GI bleeding and anemia. While other cases present with life-threatening GI bleeding that requires hemodynamic stabilization and urgent management with aggressive fluid therapy and blood transfusions [19]. On the other hand, duodenal hemangioma could also present with signs and symptoms of intestinal obstruction, intestinal perforation, and intussusception [7].

The differential diagnosis of duodenal hemangioma is wide and includes the whole differential of upper GI bleeding. Thus, the implementation of a comprehensive history and physical examination, as well as radiological imaging and endoscopy, is crucial for differentiating between the various etiologies and reaching a final diagnosis [20].

One of the important modalities utilized for diagnosing duodenal hemangiomas is upper endoscopy which provides a gross and microscopic visualization of the lesion, as well as therapeutic options including hemostatic clipping, endoscopic sclerotherapy, end loop snare, and endoscopic mucosal resection [8,21]. As shown in the current case, abdominal CT scan with IV contrast can also show a focal polypoidal non-enhancing wall thickening at the second part of the duodenum with reactive lymph node involvement. Capsule endoscopy (CE) and balloon-assisted enteroscopy can also be used to diagnose hemangiomas located in the distal small intestine, as those lesions cannot be visualized using upper endoscopy.

The mainstay therapy for duodenal hemangiomas after stabilization is open or laparoscopic surgical resection. Poor surgical candidates and patients with active GI bleeding typically undergo upper endoscopy with electrocoagulation, sclerotherapy, endoscopic mucosal resection (EMR), or argon plasma coagulation (APC) [19]. Current guidelines suggest that patients with upper GI bleeding should be initially managed with endoscopic therapy prior to surgery if a source of active bleeding is found [19]. With the advancement of endoscopic therapeutic interventions, less invasive procedures are becoming more widely employed [18]. Thus, surgical treatment of proximal small intestine bleeding is generally regarded as a last resort [19]. Of note, the use of EMR in the treatment of duodenal hemangioma has been associated with a risk of intestinal perforation. This is because intestinal hemangiomas typically originate from the deep submucosal layer, increasing the risk of bowel wall perforation. The risk of perforation is also augmented by the increased size and depth of the lesion. In our case, upper endoscopy revealed a mass in the duodenum but no source of bleeding was identified. A team of surgeons and interventional gastroenterologists decided that surgical resection of the mass was the best therapeutic approach, especially in the context of nodal involvement and a suspicious liver lesion on abdominal imaging.

It is important to point out that the differential diagnosis for our case is broad, including more common etiologies of upper GI bleeding such as peptic ulcer disease, esophagitis, gastritis, varices, and angiodysplasia among other causes [22]. This highlights the importance of including duodenal hemangioma in the differential of upper GI bleeding in cases where no obvious source of bleeding in the esophagus or the stomach is identified during endoscopy.

## Conclusions

Duodenal hemangioma is a rare cause of upper GI bleeding that commonly presents with non-specific signs and symptoms, often rendering the diagnosis delayed and difficult. Comprehensive clinical and radiological investigations, as well as microscopic examinations, are often needed to establish a definitive diagnosis. The diagnostic and therapeutic utility of minimally invasive procedures such as endoscopy makes it a preferable choice in the management of duodenal hemangiomas in a specific subset of patients. However, large lesions, as well as those with transmural involvement, and/or those with nodal and extra-nodal involvement, often prompt the need for open or laparoscopic surgical intervention.
